# Microsatellite evolution: Mutations, sequence variation, and homoplasy in the hypervariable avian microsatellite locus *HrU10*

**DOI:** 10.1186/1471-2148-8-138

**Published:** 2008-05-09

**Authors:** Jarl A Anmarkrud, Oddmund Kleven, Lutz Bachmann, Jan T Lifjeld

**Affiliations:** 1 National Centre for Biosystematics, Natural History Museum, University of Oslo, P.O. Box 1172 Blindern, NO-0318 Oslo, Norway

## Abstract

**Background:**

Microsatellites are frequently used genetic markers in a wide range of applications, primarily due to their high length polymorphism levels that can easily be genotyped by fragment length analysis. However, the mode of microsatellite evolution is yet not fully understood, and the role of interrupting motifs for the stability of microsatellites remains to be explored in more detail. Here we present a sequence analysis of mutation events and a description of the structure of repeated regions in the hypervariable, pentanucleotide microsatellite locus *HrU10 *in barn swallows (*Hirundo rustica*) and tree swallows (*Tachycineta bicolor*).

**Results:**

In a large-scale parentage analysis in barn swallows and tree swallows, broods were screened for mutations at the *HrU10 *locus. In 41 cases in the barn swallows and 15 cases in the tree swallows, mutations corresponding to the loss or gain of one or two repeat units were detected. The parent and mutant offspring alleles were sequenced for 33 of these instances (26 in barn swallows and 7 in tree swallows). Replication slippage was considered the most likely mutational process. We tested the hypothesis that *HrU10*, a microsatellite with a wide allele size range, has an increased probability of introductions of interruptive motifs (IMs) with increasing length of the repeated region. Indeed, the number and length of the IMs was strongly positively correlated with the total length of the microsatellite. However, there was no significant correlation with the length of the longest stretch of perfectly repeated units, indicating a threshold level for the maximum length of perfectly repeated pentanucleotide motifs in stable *HrU10 *alleles. The combination of sequence and pedigree data revealed that 15 barn swallow mutations (58%) produced alleles that were size homoplasic to other alleles in the data set.

**Conclusion:**

Our results give further insights into the mode of microsatellite evolution, and support the assumption of increased slippage rate with increased microsatellite length and a stabilizing effect of interrupting motifs for microsatellite regions consisting of perfect repeats. In addition, the observed extent of size homoplasy may impose a general caution against using hypervariable microsatellites in genetic diversity measures when alleles are identified by fragment length analysis only.

## Background

Microsatellites consist of tandemly repeated sequence motifs, no more than 6 bases long. They are scattered throughout most eukaryotic genomes and are extensively used as tools for a wide range of applications, such as e.g. molecular forensics, parentage testing, analysis of genetic structure of populations and the assessment of phylogenetic relationships [1]. The major characteristic that makes microsatellites a useful and powerful genetic tool is the extensive length polymorphism that first of all reflects allelic variation in the number of the tandemly arranged perfect repeats [2]. However, "interrupting motifs" (IMs) that deviate in sequence from the repeated motif and mutations in the flanking regions may also contribute to the observed length polymorphism [3].

The molecular mechanisms for the development of microsatellite variation are not completely understood. In general, microsatellites have a high mutation rate (10^-2^–10^-6^) as compared to point mutations in coding genes [4]. It is accepted that the most common mutational mechanism affecting microsatellites is replication slippage, a process involving a gain or contraction of one or more repeat units [5,6]. Other microsatellite mutations might be caused by unequal crossing over, nucleotide substitutions, or duplication events [7]. Many factors might be important for the mutational processes in microsatellites, such as e.g. allele size, motif size, gender, and G/C content [8-15]. Mutation patterns may also depend on the genomic context such as the particular location on a chromosome and functional potential of the transcribed product [9,16-18], as well as the effectiveness of mismatch repair enzymes [19,20]. Moreover, mutation rates in microsatellites are also affected by stabilization patterns and potential secondary structures [13,21].

Various models have been put forward to explain and predict the mutation processes that affect microsatellite evolution (reviewed in [22]). The infinite allele model (IAM) [23] assumes that microsatellite mutations may create an infinite number of repeated units and allelic states not present in the population. Under the stepwise mutation model (SMM) [24] microsatellite mutations have the same probability of gaining or contracting one repeat unit. Thus, this model also takes into account back mutations. The generalized stepwise model (GSM) or two phase model (TPM) is an extension of the SMM and considers the probability for a microsatellite mutation to involve more than one unit [25]. According to the K-allele model (KAM) [26] there are K allelic states and equal probabilities to mutate towards any of the other (K-1) alleles.

Most studies concerning mutational processes in microsatellites have focused on size variation among the alleles (electromorphs), and not sequence variation. Genetic approaches lacking sequence data may hide essential information, e.g. substitutions, patterns of IMs and size homoplasy [3]. Homoplasic microsatellite alleles are alleles similar in state (length), but different in descent. Accordingly, one can divide microsatellite homoplasy into two types: (1) microsatellite alleles identical in length, but not in sequence (indistinguishable by fragment length analyses), and (2) alleles identical in both length and sequences, but with different evolutionary history (only detectable through mutations documented in known pedigrees). Some theoretical studies have tried to address the impact of homoplasy on genetic diversity analyses. Navascues and Emerson [27] showed that homoplasy affects various theoretical models differently. Simulations with high mutation rates (≥10^-4^) for chloroplast microsatellites for example indicated an underestimation of homoplasy. In contrast, Estoup et al. [22] concluded that size homoplasy is not a substantial problem for population genetic studies, except for highly mutable microsatellites with strong allele size constraints in large populations.

Gaining empirical evidence of mutational processes affecting mutational diversity within natural populations is a demanding task. This is especially true for non-model organisms with comparatively few markers available and long generation time. One promising approach to overcome these difficulties is to genotype a large number of individuals in a population with known pedigree, using genetic markers with high mutation rates. One such marker is the microsatellite locus *HrU10 *[28] in the European barn swallow (*Hirundo rustica*). This marker has been mapped to chromosome 18 in the chicken (*Gallus gallus*) genome [29], yet nothing is known about its functional potential. By means of fragment length analysis, Brohede et al. [30] estimated a mutation rate of 1.56% (i.e. 15 mutations in 960 meiotic events) for this locus. Currently there is only one *HrU10 *sequence retrievable from GenBank (Accession nr: X97562). This sequence consists of a long tandemly repeated pentanucleotide (5'-TTCTC-3') stretch followed by an IM of two T's, two further repeat units and a variable tail of other arrangements of pyrimidines. Fragment length analysis in European barn swallows revealed that the majority of the *HrU10 *alleles are approximately of similar length as the *HrU10 *sequence in GenBank [30]. However, some alleles were up to three times as long. These longest *HrU10 *alleles correspond to roughly 100 tandemly repeated pentanucleotide units, a remarkably large size for a microsatellite [31]. Wierdl et al. [32] suggested microsatellite stability to be related to the length of the stretch of tandem repeats, and postulated that large microsatellites have an increased probability to realign in a misaligned confirmation during replication resulting in a higher mutation rate. Since then, increased instability of long microsatellites have been confirmed for several microsatellite loci, including *HrU10 *[30]. One may assume that, in comparison to a microsatellite with a shorter repeat motif, a pentanucleotide repeat may establish a larger spatial conformation during loop formation of a slippage event. Somewhat misaligned nucleotides might occur and further increase the probability for mutations. Thereby also IMs may be introduced.

The fragment length analysis by Brohede et al. [30] indicated several size classes of the *HrU10 *alleles in European barn swallow. Because of the high mutation rate, the *HrU10 *microsatellite locus is well suited for testing the hypotheses of longer microsatellites being more unstable and more likely to gain IMs. Accordingly, one expects a positive correlation between the number of IMs and allele length and an upper size limit for the number of perfectly repeated motifs. In the present study, we sequenced a subset of *HrU10 *alleles of different size classes from North American barn swallow and tree swallow (*Tachycineta bicolor*) that could be related to mutations in pedigree analyses [33]. This approach provides sequences from both the parent and the mutant offspring, and allows to investigate the formation of homoplasic alleles and to estimate the order of magnitude of size homoplasy for the *HrU10 *microsatellite locus.

## Methods

### Samples and Genetic Analyses

This study was based on samples previously collected for the purpose of paternity testing in Canadian populations of barn swallows and tree swallows. Both species are socially monogamous passerine birds, but with high levels of extrapair paternity [33,34]. The barn swallow samples consisted of those already reported by Kleven et al. [33] and additional samples collected during 2003 and 2004. Barn swallows were genotyped with six to nine polymorphic microsatellite markers, including *HrU10*, and a detailed description of the markers, their variability and the paternity determination are presented elsewhere [33].

Tree swallows were genotyped with three polymorphic microsatellite markers, including *HrU10*. In cases where one of the three markers showed an allelic mismatch between offspring and one of the putative parents, an additional triplet of microsatellite markers were analyzed to distinguish mutation events from extrapair paternity. Details about the microsatellite markers, their polymorphism and the parentage determination of tree swallows are provided as additional file [see Additional file 1].

Mutations were detected by comparing the genotype of the offspring with that of its biological parents. We only included individuals for whom the genotypes of both biological parents were available. Furthermore, to avoid the problem of non-amplifying alleles, we only included mutations in parents that were heterozygous at the *HrU10 *locus. We assumed the smallest mutational change in allele size to be most likely in cases where more than one parental allele could be the progenitor allele. To verify observed mutation events, we amplified microsatellite fragments twice for the parents and offspring involved in these cases.

### Sequencing

The *HrU10 *locus was amplified according to the protocol described in [33]. In addition, the reverse primer HrU10-EXT-R2 (5'-GCTGCTGTTCGAGGAAATAA-3') was designed to improve sequencing of tree swallow alleles. To reduce time consumption and lab costs we optimized a simple isolation strategy for *HrU10 *alleles that did not require cloning. Alleles with size differences > 10 base pairs (bp) were separated on MetaPhor^® ^agarose gels (Cambrex, East Rutherford, NJ) or on standard SeaKem^® ^LE Agarose (Cambrex) if the alleles differed > 50 bp in size. The allele of interest was subsequently purified with the Nucleospin^® ^Extract II gel extraction Kit (Macherey-Nagel, Düren, Germany), and sequenced directly in both directions using the BigDye Terminator v3.1 Cycle Sequencing Kit (Applied Biosystems, Foster City, CA) according to the manufacturer's recommendations on an ABI 3100 Genetic Analyser (Applied Biosystems). As a further control, the sequence lengths were compared to previous fragment length analysis of the microsatellite [33].

## Results

### Microsatellite Structure Uncovered by Sequence Analyses

Pedigree analysis based on 2076 meiotic events in barn swallows and 496 meiotic events in tree swallows revealed 41 mutations for the microsatellite locus *HrU10 *in barn swallows and 15 in tree swallows. According to fragment length analyses all mutations involved a gain or loss of five or ten bp, i.e. equivalent to one or two repeat units, which is consistent with the assumption of replication slippage. This represents a slippage rate of 1.97 × 10^-2 ^in barn swallows and 3.02 × 10^-2 ^in tree swallows. No other types of indels were observed. Sixty-six *HrU10 *alleles involved in the observed germ line mutation were sequenced, that is, 33 sets of the offspring and the donor parent (GenBank accession numbers EU295565–EU295630). There were no site (four colonies) or year (two years) effects on mutation rates in barn swallows (both *P *>0.1). The tree swallows were only sampled for one year at one location.

Woodruff et al. [35] approached the issue of "clustered mutations", which implies that related individuals may inherit identical genetic changes, contrasting an assumption that mutations are independent events. In this respect six of the parental individuals gave rise to two mutations in the same family in the barn swallow population. In these cases, identical lengths where observed only twice. Only one of these two incidents of identical mutant lengths gave adequate sequences of both mutants. Nevertheless, in the particular case (Mut12 and Mut18 [see Additional file 2]) where sequences were obtained from both mutants, the outcome of the two mutations was dissimilar, verifying that the mutations represent independent events.

The nucleotide sequences confirmed that there was a gain or a contraction of one or two pentanucleotide units in all the 33 germ lines (26 in barn swallows and seven in tree swallows [see Additional file 2]). No nucleotide substitutions or other indels were detected. The sequence data revealed several different IMs for the *HrU10 *alleles in both species, all of which consisted of distinct rearrangements of 1–30 pyrimidine bases (T and C). No purine bases were detected in the pyrimidine rich strand of the *HrU10 *microsatellite. The subsequent statistical analyses were performed on the barn swallow parental alleles only, as the number of alleles that gave adequate sequences in tree swallows was considered too low (*n *= 7) for statistical testing.

**Figure 1:**
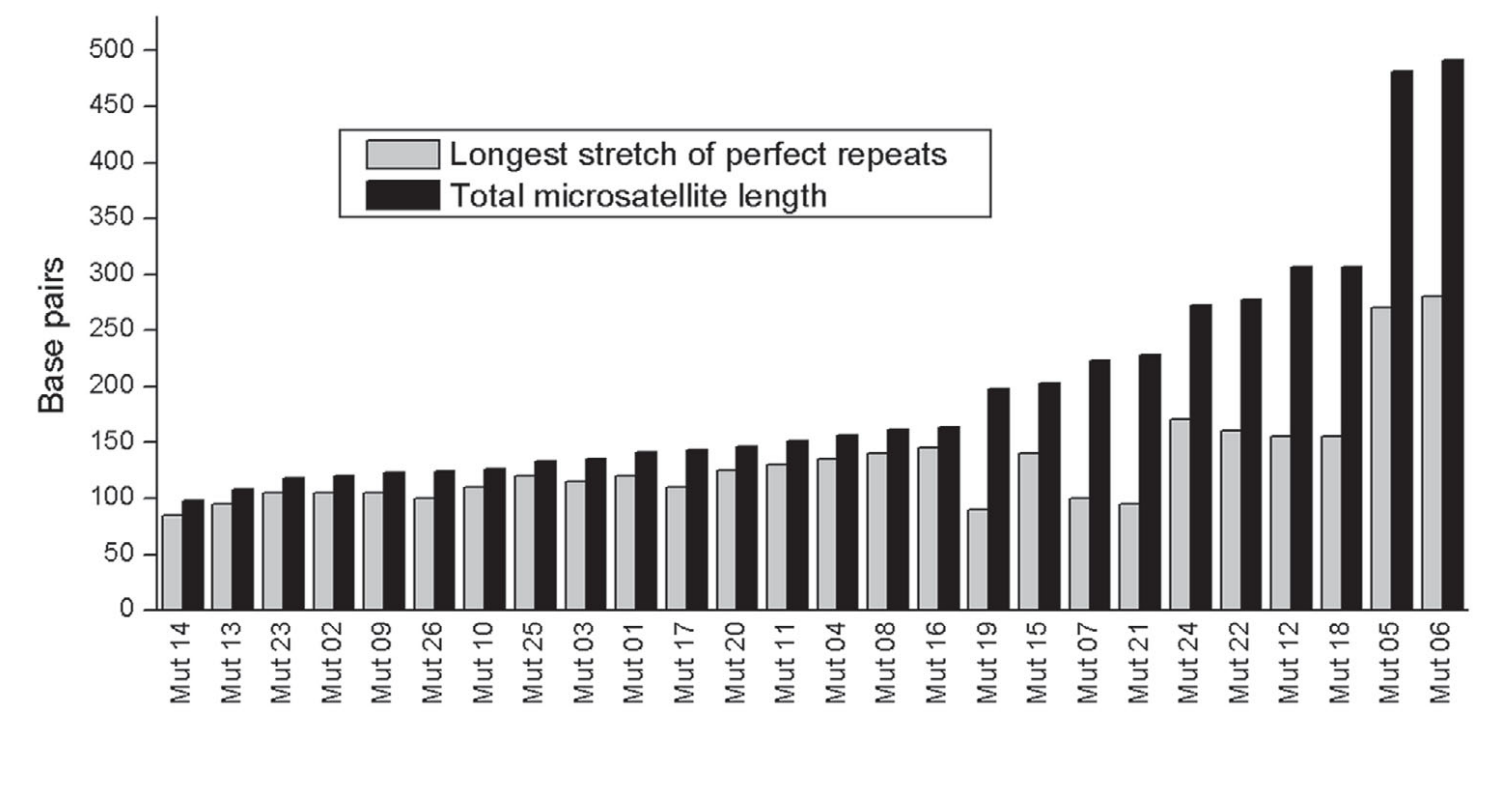
**Correlation of array length of perfect repeats and *HrU10 *microsatellite length**. Plot of the longest stretch of perfectly repeated units and the corresponding total *HrU10 *microsatellite length in sequenced mutant barn swallow alleles.

First, we tested the hypothesis that longer microsatellites are more unstable and will consequently contain more IMs. If such a correlation occurred, then one would expect an upper size limit for the length of perfect repeats, and, accordingly there should be no correlation between the length of the longest stretch of perfect repeats of a given *HrU10 *allele and the number of IMs. Correlation tests between total microsatellite length and (1) number of IMs (Spearman: r_s _= 0.55, *P *= 0.003), and (2) total number of nucleotides contributing to the IMs (r_s _= 0.54, *P *= 0.004) were significant. No significant correlation was found between the longest stretch of perfect tandemly repeated units and (1) number of IMs (r_s _= 0.09, *P *= 0.66) or (2) total number of nucleotides in the IMs (r_s _= 0.14, *P *= 0.5) (Figure 1).

We further determined roughly in which part of the *HrU10 *allele a slippage event had taken place. The 16 shortest alleles showed one long (17–29 units) and one or two short (1–2 units) stretches of perfectly repeated units that are separated by IMs. The 10 longest alleles showed two long (14–55 units) and up to three short (0–5 units) stretches of perfectly repeated units. All replication induced mutations could be attributed to the long (≥14 units) stretches of perfect repeats. In 21 of 26 alleles (81%), the slippage mutation affected the longest stretch of perfectly repeated units. It is noteworthy that the other 5 mutations that did not occur in the longest stretch of perfect repeats, all affected the alleles that were among the 10 longest.

In the barn swallow, 15 of the 26 (58%) observed mutations resulted in mutant alleles that were homoplasic to another sequenced allele in the data set. Nine (35%) of these mutations resulted in homoplasic alleles with respect to length, and six (23%), mutant alleles were also identical in sequence to another allele. No incidents of homoplasy due to mutations were detected in the tree swallows, but the number of sequences was significantly lower.

### Features of the Mutations Revealed by the Fragment Length Analyses

**Figure 2:**
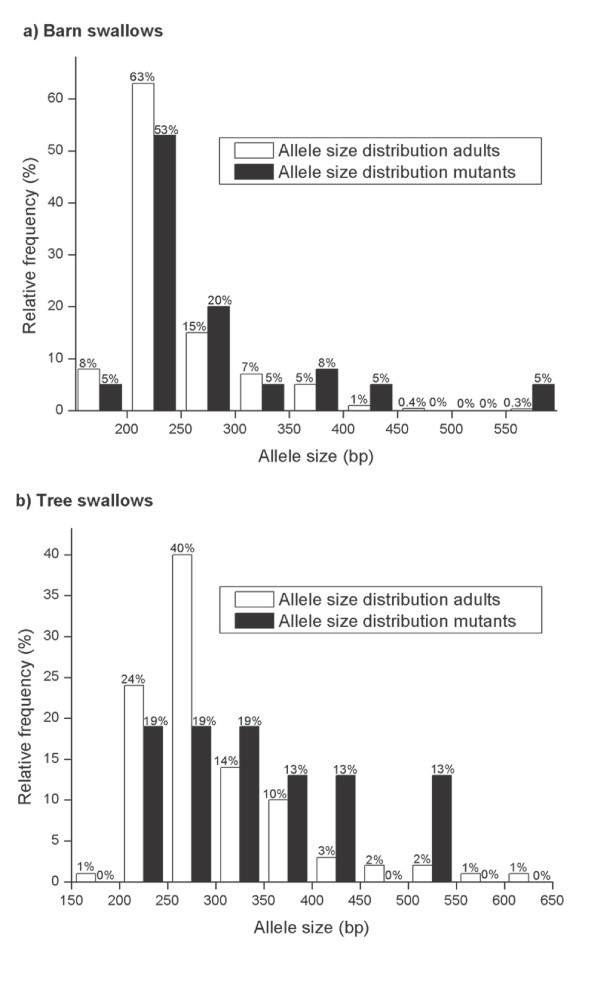
**Size distribution of mutant and parental *HrU10 *allele sizes**. Size distribution of the *HrU10 *locus in the adult population (white bars) and the mutant alleles (black bars) in **a) **barn swallows (*n *= 375) and **b) **tree swallows (*n *= 144). Each bar represents the alleles from the corresponding size class, which has been organized in groups of 50 and 50 bp.

**Figure 3:**
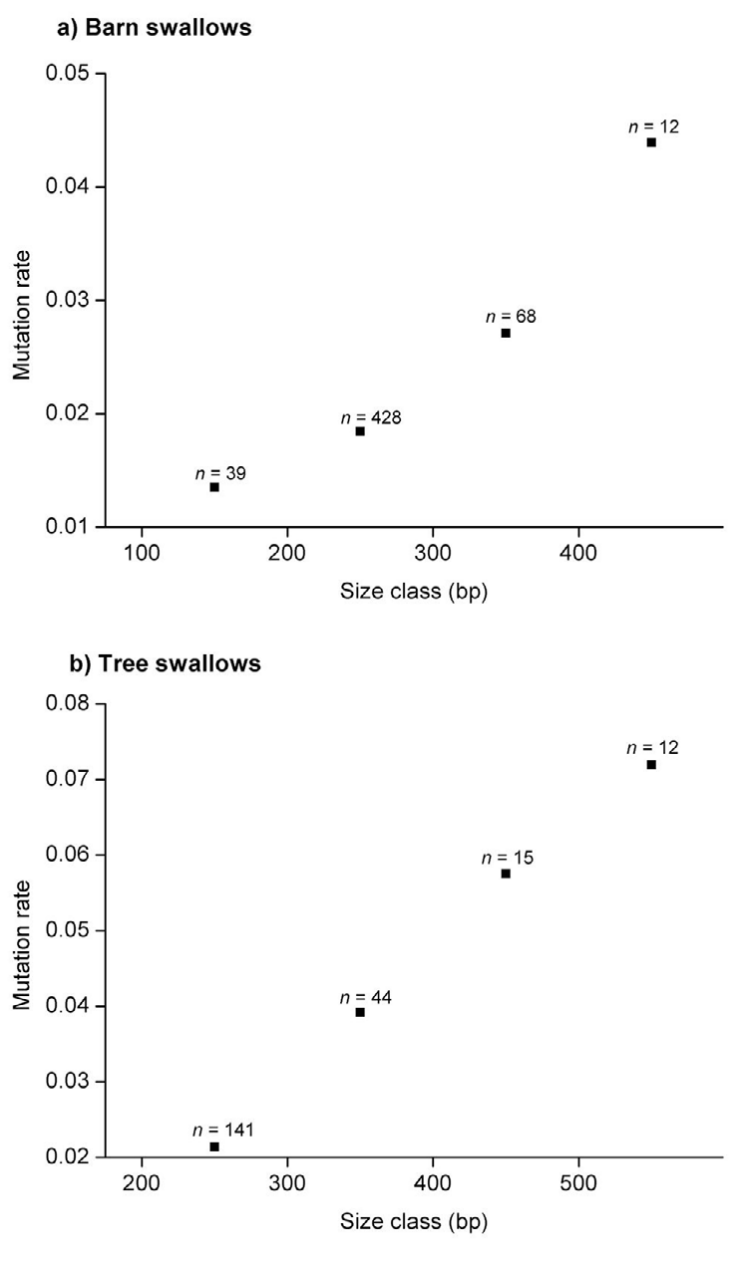
**Correlation between mutation rate and *HrU10 *allele sizes**. Relationship between mutation rate and length of genotyped alleles in the population of biological parents in **a) **barn swallows and **b)** tree swallows. The alleles were lumped into 4 size classes (barn swallows: class 1 = 100–199 bp, class 2 = 200–299 bp, class 3 = 300–399 bp, class 4 = 400+ bp; tree swallows: class 1 = 200–299 bp, class 2 = 300–399 bp, class 3 = 400–499 bp, class 4 = 500+ bp). *n *refers to number of alleles in the particular size class.

The genotyped adult population consisted of 376 and 144 individuals in the barn and tree swallow populations, respectively. The allele size frequencies for the *HrU10 *locus in the two species are illustrated in Figure 2 (note: 95 bp in the flanking regions of the microsatellite are not included in the presented sequences but in the fragment analyses). Due to seven non-amplifying alleles, only 745 barn swallow alleles were included. The median allele size of the *HrU10 *microsatellite in the adult barn swallow population was 231 bp (± 2.0 SE, range = 175–581 bp, *n *= 704 alleles). Median size of the microsatellite of the mutant barn swallow alleles was 241 bp (± 13.8, range = 193–586, *n *= 41), i.e. significantly longer than for the entire population (Mann-Whitney U test: Z = -2.4, *P *= 0.02). The median allele size for the *HrU10 *locus in the tree swallow population was 280 bp (± 4.6, range = 186–605, *n *= 272 alleles), and the median microsatellite length of the mutant tree swallow alleles was 323 bp (± 24.5, range = 222–518, *n *= 15). The mutation rates showed a tendency to be positively correlated with allele sizes for both barn swallows (GLM with binominal error distribution and logit link: χ^2^_1 _= 9.26, *P *= 0.002) and tree swallows (GLM with binominal error distribution and logit link: χ^2^_1 _= 3.35, *P *= 0.067). Estimations of mutation rates in relation to allele sizes are illustrated in Figure 3.

There were 26 (65%) expanding and 14 (35%) contracting mutations in the barn swallow population, which was not significantly different from equity (binominal test (two-tailed): *P *= 0.08), but may indicate a bias toward expansion. Directionality of the mutation was impossible to determine in one case because of a 5 bp difference to both possible parental alleles. No indication of directional bias for *HrU10 *mutations was found in tree swallows with 7 (47%) expansions and 8 (53%) contractions (binominal test: *P *= 1.0). Directionality of mutations were not significantly affected by allele size neither in barn swallows (GLZ with binomial error distribution; *χ*^2 ^= 0.2, *P *= 0.7) nor in tree swallows (*χ*^2 ^= 0.1, *P *= 0.78). Furthermore, there was no positive correlation between length of the longest perfectly repeated core microsatellite unit and the directionality of the mutations in barn swallows (GLZ with binomial error distribution; *χ*^2 ^= 0.1, *P *= 0.81).

In both species, there was a tendency for mutations to be maternally transmitted, as 66% (27/41) of the barn swallow mutations were observed in the female germ line (binominal test: *P *= 0.06) and 80% (12/15) in tree swallows (binominal test: *P *= 0.035).

## Discussion

The sequence analyses presented in this study document two major features of the *HrU10 *locus: (1) Frequent introductions of IMs which were strongly positively correlated with allele size and (2), frequent generation of homoplasic alleles (>50% of the mutations in the sequenced data set).

### Evolution of *HrU10 *– Introductions of IMs and Mutational Patterns

All IMs consisted of different arrangements of Cs and Ts. No introduction of purines on the pyrimidine-rich strand was observed. The repeated motif of the *HrU10 *locus is a pentanucleotide, and its spatial conformation during a slippage event may involve a larger loop compared to a smaller repeat unit. This may indicate that all the *HrU10 *IMs are results of uncompleted replication slippage. If so, one needs to assume that the loop on the nascent or template strand will not include an entire unit before it realigns. This hypothesis is in line with all the IMs detected for *HrU10*, except for the (TTCCC)_6_-repeat in Mut5 and Mut6 [see Additional file 2], which happened to be the longest *HrU10 *alleles sequenced in the barn swallows. These two mutants have originated from the same parental allele that is 3.5 times longer than the median allele length.

An interesting feature related to the IMs, is the relatively constant maximal length of stretches of perfectly repeated core units, indicating a threshold length for stable arrays of perfectly repeated microsatellite units (Figure 1). One explanation for such a threshold level may be a selection pressure to retain stabile conformations. Such a selection pressure has been suggested to be important for preserving folding potential in repeated di-nucleotides [21,36] and tri-nucleotides [37]. In this respect, nothing is currently known for pentanucleotides.

Because the longest stretch of perfectly repeated units contributes most to the total microsatellite length in the *HrU10 *alleles with only one long stretch of perfect tandem repeats, it is not surprising that the slippage events were restricted to this region of the microsatellite. Furthermore, theoretical and experimental approaches have suggested that slippage rates are approximately zero in very short repeated regions [38-41], supporting a low probability for slippages to occur in the short stretches in the *HrU10 *alleles. Nevertheless, if length of perfectly repeated tandem units is more crucial for microsatellite instability than total microsatellite length, one would predict a consistent bias for slippage to affect the longest stretch of alleles comprising at least two long (>14 units) stretches of perfect core units. However, only 50% (5/10) of the mutations were introduced in the longer of the two stretches. This result is in agreement with a theory of total length of entire microsatellite being a more important factor for microsatellite stability than longest motif of perfect tandemly units. The observed threshold level for length of perfectly repeated units before introductions of IMs might then be explained by incomplete slippage (described above) being an increasingly more important mutational mechanism as the allele is destabilized due to growth. However, the statistical power for such a conclusion is relatively poor. It is noteworthy that an opposite pattern was detected in an *in vitro *system of mono- and dinucleotides in a mutation study of human cell lineages [38]. Selection pressure for the maintenance of IMs has also been proposed important for tri-nucleotide repeat stability in genes related to various types of spinocerebellar ataxia [16,37,42].

The results from the fragment length analyses also support the prediction of long *HrU10 *alleles to be more unstable, as the probability of a slippage event was positively related to allele sizes. The mutation rates were estimated to be more than three-fold higher in the longest compared with the smallest allele classes in the two swallow populations (Figure 3). Higher mutation rates for longer repeated regions have been reported in a number of previous studies [5,10,11,30,32,36,38,39,43-46]. The higher mutability in longer alleles can be explained by stabilization patterns concerning the mismatch-repair system, which may be less effective and as a result generate a relatively large probability for insertion of slippage events if the repeated region is sufficiently long, as suggested by Wierdl et al. [32]. The logic in this theory is that the number of possible conformations increases proportionally with the increase of repeated microsatellite units. However, if this hypothesis was correct, the microsatellite could, in theory, obtain uncontrolled growth and finally strike a large region on the chromosome. Nevertheless, microsatellites seems to have an upper size limit that rarely exceed 50 repeated units [31]. Still, some of the *HrU10 *alleles uncovered in our study include almost 100 pentanucleotide units.

It has been suggested that short microsatellites tend to gain additional units whereas long microsatellites are more likely to lose units during a mutation event [39,47]. However, the support for this hypothesis is ambiguous. Primmer et al. [11], Eckert et al. [13] and Vigouroux et al. [48] have reported an overall directionality bias of slippage leading to expansion. Our data showed a similar tendency for barn swallow, though not statistically significant. Xu et al. [49] showed a constant slippage rate for expansion and an increasing slippage rate for contraction with increasing allele size, which is consistent with a general observation of an upper size limit for repeated regions. However, our data on the *HrU10 *locus do not support that slippage directionality is length dependent, neither for total length of perfectly repeated core units nor for the total length of the entire microsatellite region. This result is in agreement with earlier studies for the *HrU10 *locus [30].

Weber and Wong [50]; Garza et al. [51]; and Primmer et al. [52] have put forward hypotheses for the upper size limit for microsatellites. They postulate that the contraction of microsatellites is caused by large deletions that occur when a microsatellite reaches its maximal length potential. An example of such large deletion has been reported by Colson and Goldstein [53] who reported one incidence of an absent microsatellite in one allele of the *U1951 *locus in *Drosophila melanogaster*. Other examples include a 27 units contraction of a dinucleotide microsatellite in *Ranunculus carpaticola *[46], and a 18 units contraction in a tetranucleotid repeat in superb fairy-wrens (*Malurus cyaneus*) [43]. It has also been suggested that if the balance between slippage and point mutations favours point mutations within the repeated region, the mutations may interrupt the feature of the microsatellite without enhancing large contractions [54], and eventually give rise to new diversity. Kruglyak et al. [55] developed a Markov chain model which confirmed that infinite microsatellite growth can be disabled by introductions of point mutations. In the sequence data presented here, all sequences contain IMs. These must have been introduced by other mutation mechanisms than regular slippage of entire units.

Because of the higher number of mitotic cell divisions in male than in female germ lines, it is plausible to expect that evolution of microsatellites, to some extent, is male-driven [14,15]. However, we observed a bias for mutations at the *HrU10 *locus to be maternally transmitted in both barn swallows and tree swallows. Similar results have been reported by Brohede et al. [30] who observed a 2.5–5 fold increase in slippage rates in several hypermutable markers in females barn swallows compared to males, including the *HrU10 *microsatellite. Beck et al. [43] also uncovered a bias favouring maternally transmitted slippages for one locus in the superb fairy-wren (*Malurus cyaneus*).

### Cases of Size Homoplasy

More than 50% of the *HrU10 *mutations sequenced in this study resulted in a mutant allele size homoplasic to another sequenced allele in the dataset, and 23% of the mutations resulted in alleles homoplasic in both allele length and sequence (identical alleles). Such homoplasy is only detectable through sequencing of observed mutations in known pedigrees and has to our knowledge not earlier been confirmed by empirical data. Size homoplasy may be problematic according to Estoup et al. [22] in instances with "(1) high mutation rates and (2) large population sizes together with (3) strong allele size constraints". The mutation rates observed in this study (1.97 × 10^-2 ^per meiosis in barn swallow, 3.02 × 10^-2 ^per meiosis in tree swallow) are among the highest ever reported for microsatellites, and our estimate is also concordant with that provided by Brohede et al. [30] for the same locus. A total number of 2070 meiotic events for the barn swallow population presented here is the largest data set ever reported for an avian pentanucleotide microsatellite. Although it seems unlikely that there is a strong allele size constraint for the *HrU10 *locus, our empirical results confirm that most of the observed mutations resulted in an electromorph already present among the 66 sequenced alleles. In consequence, homozygous individuals in terms of fragment length analyses are indeed not necessarily homozygous in terms of nucleotide sequences. Discrepancies between allelic variation detectable through fragment length analyses and sequence analyses have also been reported in other microsatellite studies [3,53,56]. Estoup et al. [22] approached the issue of size homoplasy theoretically based on frequently applied mutations models such as e.g. the SMM and the KAM. The proposed index of size homoplasy can be explained as the probability of two electromorphic alleles not being of common descent. However, these indexes have certain limitations when applied to the data on the *HrU10 *locus provided here. First, the proposed homoplasy estimates relates to length and not sequence. Accordingly, there is no parameter that distinguishes between the two types of homoplasy. Second, the homoplasy index for the SMM does not include a parameter for the number of allelic states in a population, which is certainly crucial for homoplasy estimates. Third, the KAM accounts for allelic states, but assumes equal probability to mutate towards any of the other K-1 alleles. This is certainly not the case for the *HrU10 *locus in barn swallows.

Many studies have focused on the occurrence of microsatellite size homoplasy within different taxa (e.g: humans (*Homo sapiens*) and chimpanzees (*Pan troglodytes*) [57], mammalian carnivores [58,59], birds [60,61], salmonids [3], pipefish (*Syngnathus typhle*) [62], crabs (*Limulus polyphemus*) [63], two bee species and the fresh water snail *Bulinus truncates *[64] and fruit flies (*Drosophila*) [65]). These examples support the notion that caution must be taken when microsatellite data are collected by electromorphic genotyping only. The high rate of mutations leading to size homoplasy in the present study provides support for alleles of identical size being attributed to common descent and hence causing bias in population genetic estimates.

Estoup et al[66] and Estoup and Cournet [67] suggested that the amount of size homoplasy is more important in interspecific than in intraspecific comparisons. Nevertheless, our results for *HrU10 *provide evidence that homoplasy may play an important role also within populations. This conclusion is in agreement with the results published by van Oppen et al. [68], who found equally high amounts of homoplasy when comparing individuals among closely related taxa and among more distantly related species. *HrU10 *is a frequently used marker for different genetic analyses of bird populations, especially parentage studies [33,69-72] because of the high allelic diversity enabling a powerful marker for parentage testing. However, our study indicates that *HrU10 *should be used with caution whenever homoplasy may cause biased estimates of relatedness and genetic diversity.

## Conclusion

Sequencing of 33 mutated and 66 parental *HrU10 *alleles was consistent with the hypotheses that longer alleles tend to be more instable due to increased slippage rate. The observed positive correlation between the number of IMs and allele size supported the assumption of a threshold level for the maximal length of stable perfect repeats. Nonetheless, the particular location of the slippage positions in the mutated microsatellite alleles indicated that total microsatellite length is more important for microsatellite stability than the length of the longest stretch of perfect repeats. Mainly because of the high slippage rate, there is also a high level of homoplasy at the *HrU10 *locus, i.e. 58% of the characterized mutations yielded an electromorph already present in the sequenced data set, including both type 1 and type 2 size homoplasy. The problem of size homoplasy imposes a general caution of using such hypermutable markers in fragment analyses assuming unique alleles by size only.

## Authors' contributions

JAA carried out the sequence analyses, interpreted results and drafted the manuscript. OK performed the field work, carried out the fragment analyses, helped designing the study and drafting the manuscript. LB helped designing the study, interpreting results and drafting the manuscript. JTL initiated the study and helped drafting the manuscript. All authors read and approved the final manuscript.

## Supplementary Material

Additional file 1 Analysis of paternity in tree swallows. A detailed description of the markers used for paternity analysis in the tree swallows. Click here for file

Additional file 2 Table presenting the sequenced *HrU10 *microsatellites. *HrU10 *microsatellite sequences detected in **a) **barn swallows and **b) **tree swallows. The underlined nucleotides represent the core microsatellite unit (nucleotides not underlined are inserts of IMs). The regions in which a mutation occurred are depicted by red numbers. "bp" refers to total numbers of base pairs in the microsatellite region and "Type" refers to numbers of gains or contractions of units each mutation caused. All mutations which resulted in electromorphic identity with another sequenced allele are listed below the table. Click here for file
