# Supplementary material for: Microsatellite evolution: Mutations, sequence variation, and homoplasy in the hypervariable avian microsatellite locus HrU10
Source: BMC Evol Biol. 2008 May 9;8:138. doi: 10.1186/1471-2148-8-138 (PMC2396632; doi:10.1186/1471-2148-8-138)
Supplement: Additional file 1 — Analysis of paternity in tree swallows. A detailed description of the markers used for paternity analysis in the tree swallows. [file 1471-2148-8-138-S1.pdf]

ELECTRONIC APPENDIX

This is the Electronic Appendix to the article

Microsatellite evolution: Mutations, sequence variation, and homoplasy in the hypervariable avian microsatellite locus *HrU10*

by

Jarl A. Anmarkrud, Oddmund Kleven, Lutz Bachmann, Jan T. Lifjeld

BMC Evolutionay Biology

Analysis of paternity in tree swallows

DNA was extracted from blood or tissue samples with a commercial kit (E.Z.N.A, Omega Bio-Tek, U.S.A.) and paternity analysed based on three highly polymorphic microsatellite markers through polymerase chain reaction (PCR). Each 10 µL reaction consisted of about 30 ng of genomic DNA, 0.5 µM of each primer (forward primers were fluorescently dyed), 0.1 mM dNTP mix (ABgene, U.K.) and 0.2 units of DNA polymerase (DyNAzyme, Finnzymes, Finland) in the manufacturer's buffer (final concentrations of 10 mM Tris HCl, 1.5 mM MgCl2, 50 mM KCl, 0.1% Triton X-100). PCR was run on a GeneAmp 9700 Thermocycler (Applied Biosystems, U.S.A.). The PCR profile used consisted of an initial denaturing step at 94°C for 5 min, followed by 35 cycles consisting of 94°C for 30 s, X°C (50°C for locus *Aar4*, and 55°C for loci *HrU6* and *HrU10*) for X s (30 s for locus *Aar4*, and 40 s for loci *HrU6* and *HrU10*), and 72°C for 30 s. The PCR profile was terminated with 72°C for 7 min followed by 4°C for 5 min. PCR products were sized using a capillary automated ABI 3100 sequencer (Applied Biosystems, U.S.A.) and the data was analyzed with GeneMapper v3.0 analytical software (Applied Biosystems, U.S.A.). Polymorphism of the microsatellite markers was calculated using CERVUS v3.0 [1] (see table 1). The combined exclusion probability for the three markers was higher than 0.99. Null-alleles were detected at one locus (*HrU10*) and mutations at two loci (*HrU6* and *HrU10*). Hence, to resolve cases of paternity uncertainty due to a single allelic mismatch between the genotype of a young and the putative parent, we used an additional triplet of microsatellite markers (*Ltr6*, *Pdoµ5* and *Tbi81*). Detailed information about the PCR protocol and polymorphism of these latter markers is presented elsewhere [2].

Table 1. Polymorphism among three microsatellite loci used for analysis of paternity in tree swallows (*Tachycineta bicolor*). Data on the number of alleles (k), number of genotyped adult individuals (n), observed heterozygosity (H_O), expected heterozygosity (H_E), probability of exclusion assuming no parents known (P_{ei}), probability of exclusion assuming one parent known (P_{ej}), and estimated frequency of null-alleles (N_e) are presented.

locus (references)	k	n	H_O	H_E	P_{ei}	P_{ej}	N_e
Aar4 [3]	15	146	0.88	0.89	0.62	0.77	+0.002
HrU6 [4]	81	146	0.92	0.92	0.71	0.83	−0.003
HrU10 [5]	142	144	0.84	0.99	0.96	0.98	+0.082

References

1. Kalinowski ST, Taper ML, Marshall TC: **Revising how the computer program CERVUS accommodates genotyping error increases success in paternity assignment**. *Mol Ecol* 2007, **16**(5):1099-1106.
2. Stapleton MK, Kleven O, Lifjeld JT, Robertson RJ: **Female tree swallows (*Tachycineta bicolor*) increase offspring heterozygosity through extrapair mating**. *Behav Ecol Sociobiol* 2007, **161**:1725-1733.
3. Hansson B, Bensch S, Hasselquist D, Lillandt BG, Wennerberg L, von Schantz T: **Increase of genetic variation over time in a recently founded population of great reed warblers (*Acrocephalus arundinaceus*) revealed by microsatellites and DNA fingerprinting**. *Mol Ecol* 2000, **9**(10):1529-1538.
4. Primmer CR, Møller AP, Ellegren H: **Resolving genetic relationships with microsatellite markers: a parentage testing system for the swallow *Hirundo rustica***. *Mol Ecol* 1995, **4**(4):493-498.
5. Primmer CR, Møller AP, Ellegren H: **New microsatellites from the pied flycatcher *Ficedula hypoleuca* and the swallow *Hirundo rustica* genomes**. *Hereditas* 1996, **124**:281-283.
