# Supplementary material for: Microsatellite evolution: Mutations, sequence variation, and homoplasy in the hypervariable avian microsatellite locus HrU10
Source: BMC Evol Biol. 2008 May 9;8:138. doi: 10.1186/1471-2148-8-138 (PMC2396632; doi:10.1186/1471-2148-8-138)
Supplement: Additional file 2 — Table presenting the sequenced HrU10 microsatellites. HrU10 microsatellite sequences detected in a) barn swallows and b) tree swallows. The underlined nucleotides represent the core microsatellite unit (nucleotides not underlined are inserts of IMs). The regions in which a mutation occurred are depicted by red numbers. "bp" refers to total numbers of base pairs in the microsatellite region and "Type" refers to numbers of gains or contractions of units each mutation caused. All mutations which resulted in electromorphic identity with another sequenced allele are listed below the table. [file 1471-2148-8-138-S2.pdf]

a) Barn swallow

Mut		Sequence	bp	Type
1	Parent	TCCTC(TTCTC)$_{24}$TT(TTCTC)$_2$(TC)$_2$	141	+1
	Offspring	TCCTC(TTCTC)$_{25}$TT(TTCTC)$_2$(TC)$_2$	146[a]	
2	Parent	TC(TTCTC)$_{21}$TTC(TTCTC)$_2$	120	+1
	Offspring	TC(TTCTC)$_{22}$TTC(TTCTC)$_2$	125	
3	Parent	(TC)$_2$(TTCTC)$_{23}$TT(TTCTC)$_2$(TC)$_2$	135	-1
	Offspring	(TC)$_2$(TTCTC)$_{22}$TT(TTCTC)$_2$(TC)$_2$	130	
4	Parent	TCCTC(TTCTC)$_{27}$TT(TTCTC)$_2$(TC)$_2$	156	-2
	Offspring	TCCTC(TTCTC)$_{25}$TT(TTCTC)$_2$(TC)$_2$	146[a]	
5	Parent	TCCTC(TTCTC)$_{32}$(TTCCC)$_6$(TTCTC)$_{55}$TT(TTCTC)$_2$(TC)$_2$	486[b]	+1
	Offspring	TCCTC(TTCTC)$_{32}$(TTCCC)$_6$(TTCTC)$_{56}$TT(TTCTC)$_2$(TC)$_2$	491[b]	
6	Parent	TCCTC(TTCTC)$_{32}$(TTCCC)$_6$(TTCTC)$_{55}$TT(TTCTC)$_2$(TC)$_2$	486[b]	-1
	Offspring	TCCTC(TTCTC)$_{32}$(TTCCC)$_6$(TTCTC)$_{54}$TT(TTCTC)$_2$(TC)$_2$	481[b]	
7	Parent	(TTCTC)$_4$TCCTC(TTCTC)$_{20}$TC(TTCTC)$_{17}$TT(TTCTC)(TC)$_2$	223[c]	+1
	Offspring	(TTCTC)$_4$TCCTC(TTCTC)$_{20}$TC(TTCTC)$_{18}$TT(TTCTC)(TC)$_2$	228[d]	
8	Parent	TCCTC(TTCTC)$_{28}$TT(TTCTC)$_2$(TC)$_2$	161	+1
	Offspring	TCCTC(TTCTC)$_{29}$TT(TTCTC)$_2$(TC)$_2$	166	
9	Parent	TC(TTCTC)$_{21}$TT(TTCTC)$_2$(TC)$_2$	123[e]	-1
	Offspring	TC(TTCTC)$_{20}$TT(TTCTC)$_2$(TC)$_2$	118[f]	
10	Parent	(TTCTC)$_{22}$TT(TTCTC)$_2$(TC)$_2$	126	-1
	Offspring	(TTCTC)$_{21}$TT(TTCTC)$_2$(TC)$_2$	121	
11	Parent	TCCTC(TTCTC)$_{26}$TT(TTCTC)$_2$(TC)$_2$	151[g]	-1
	Offspring	TCCTC(TTCTC)$_{25}$TT(TTCTC)$_2$(TC)$_2$	146[a]	
12	Parent	(TTCTC)$_{31}$(TTCCTC)(TTCTC)$_{26}$TT(TTCTC)$_2$(TC)$_2$	307[h]	+1
	Offspring	(TTCTC)$_{31}$(TTCCTC)(TTCTC)$_{27}$TT(TTCTC)$_2$(TC)$_2$	312[i]	
13	Parent	(TTCTC)$_{19}$TTC(TTCTC)$_2$	108	-1
	Offspring	(TTCTC)$_{18}$TTC(TTCTC)$_2$	103[l]	
14	Parent	(TTCTC)$_{17}$TTC(TTCTC)$_2$	98	+1
	Offspring	(TTCTC)$_{18}$TTC(TTCTC)$_2$	103[l]	
15	Parent	TCCTC(TTCTC)$_5$TTC(TTCTC)$_{28}$TTCT(TTCTC)$_2$TT(TTCTC)$_2$(TC)$_2$	203[j]	-1
	Offspring	TCCTC(TTCTC)$_5$TTC(TTCTC)$_{27}$TTCT(TTCTC)$_2$TT(TTCTC)$_2$(TC)$_2$	198[k]	
16	Parent	TC(TTCTC)$_{29}$TT(TTCTC)$_2$(TC)$_2$	163	+1
	Offspring	TC(TTCTC)$_{30}$TT(TTCTC)$_2$(TC)$_2$	168	
17	Parent	(TTCTC)$_{22}$TT(TTCTC)$_3$TC(TTCTC)(TC)$_2$	138[o]	+1
	Offspring	(TTCTC)$_{23}$TT(TTCTC)$_3$TC(TTCTC)(TC)$_2$	143	
18	Parent	(TTCTC)$_{31}$(TTCCTC)(TTCTC)$_{26}$TT(TTCTC)$_2$(TC)$_2$	307[h]	+1
	Offspring	(TTCTC)$_{32}$(TTCCTC)(TTCTC)$_{26}$TT(TTCTC)$_2$(TC)$_2$	312[i]	
19	Parent	TCCTC(TTCTC)$_{14}$TTC(TTCTC)$_{18}$TTCT(TTCTC)$_2$TT(TTCTC)$_2$(TC)$_2$	198[k]	+1
	Offspring	TCCTC(TTCTC)$_{15}$TTC(TTCTC)$_{18}$TTCT(TTCTC)$_2$TT(TTCTC)$_2$(TC)$_2$	203[j]	
20	Parent	TCCTC(TTCTC)$_{25}$TT(TTCTC)$_2$(TC)$_2$	146[a]	+1
	Offspring	TCCTC(TTCTC)$_{26}$TT(TTCTC)$_2$(TC)$_2$	151[g]	
21	Parent	(TTCTC)$_4$TCCTC(TTCTC)$_{19}$TC(TTCTC)$_{18}$TT(TTCTC)$_2$(TC)$_2$	228[d]	+1
	Offspring	(TTCTC)$_4$TCCTC(TTCTC)$_{19}$TC(TTCTC)$_{17}$TT(TTCTC)$_2$(TC)$_2$	223[c]	
22	Parent	(TTCTC)$_{23}$TC(TTCTC)$_{32}$	277	+1
	Offspring	(TTCTC)$_{24}$TC(TTCTC)$_{32}$	282	
23	Parent	(TTCTC)$_{21}$TTC(TTCTC)$_2$	118[f]	+1
	Offspring	(TTCTC)$_{22}$TTC(TTCTC)$_2$	123[e]	
24	Parent	(TTCTC)$_{20}$TC(TTCTC)$_{34}$	272	+1

	Offspring	(TTCTC)$_{20}$TC(TTCTC)$_{35}$	277	
25	Parent	(TTCTC)$_{24}$TTC(TTCTC)$_{2}$	133	+1
	Offspring	(TTCTC)$_{25}$TTC(TTCTC)$_{2}$	138[o]	
26	Parent	TCCTC(TTCTC)$_{20}$TTCTT(TTCTC)$_{2}$(TC)$_{2}$	124	-1
	Offspring	TCCTC(TTCTC)$_{19}$TTCTT(TTCTC)$_{2}$(TC)$_{2}$	119	

[a] Size homoplasy, identical alleles (Mut1-, Mut4-, Mut11-offspring and Mut20-parent).
[b] Mut5- and Mut6-offspring share parental allele that has mutated, although different outcome of the mutations.
[c] Size homoplasy, heterogenic states (between Mut7-parent and Mut21-offspring).
[d] Size homoplasy, heterogenic states (between Mut7-offspring and Mut21-parent).
[e] Size homoplasy, heterogenic states (between Mut9-parent and Mut23-offspring).
[f] Size homoplasy, heterogenic states (between Mut9-offspring and Mut23-parent).
[g] Size homoplasy, identical alleles (Mut11-parent and Mut20-offspring).
[h] Mut12- and Mut18-parent, the same allele which has mutated and resulted in [i].
[i] Size homoplasy, heterogenic states (see [h]).
[j] Size homoplasy, heterogenic states (between Mut15-parent and Mut19-offspring).
[k] Size homoplasy, heterogenic states (between Mut15-offspring and Mut19-parent).
[l] Size homoplasy, identical alleles (Mut13- and Mut14-offspring).
[o] Size homoplasy, heterogenic states (between Mut17-parent and Mut25offspring)

b) Tree swallow

**Mut**		**Sequence**	**BP**	**Type**
1	Parent	CTC(TTCTC)$_{2}$TC(TTCTC)$_{19}$(TC)$_{2}$(TTCTC)$_{3}$TT(TTCTC)TC(TTCTC)	143	-2
	Offspring	CTC(TTCTC)$_{2}$TC(TTCTC)$_{17}$(TC)$_{2}$(TTCTC)$_{3}$TT(TTCTC)TC(TTCTC)	133	
2	Parent	TTC(TTCTC)$_{2}$TC(TTCTC)$_{42}$C	226	+1
	Offspring	TTC(TTCTC)$_{2}$TC(TTCTC)$_{43}$C	231	
3	Parent	CTC(TTCTC)$_{25}$TT(TTCTC)TCT(TTCTC)TC(TTCTC)	150[m]	-1
	Offspring	CTC(TTCTC)$_{24}$TT(TTCTC)TCT(TTCTC)TC(TTCTC)	145[n]	
4	Parent	CTC(TTCTC)$_{25}$TT(TTCTC)TCT(TTCTC)TC(TTCTC)	150[m]	-1
	Offspring	CTC(TTCTC)$_{24}$TT(TTCTC)TCT(TTCTC)TC(TTCTC)	145[n]	
5	Parent	(TC)$_{2}$(TTCTC)$_{32}$TTC(TTCTC)TTTC	176	+1
	Offspring	(TC)$_{2}$(TTCTC)$_{33}$TTC(TTCTC)TTTC	181	
6	Parent	(TC)$_{3}$TT(TTCTC)$_{2}$TT(TTCTC)$_{18}$(TCTTCTC)$_{6}$TC(TTCTC)$_{31}$C	310	+1
	Offspring	(TC)$_{3}$TT(TTCTC)$_{2}$TT(TTCTC)$_{18}$(TCTTCTC)$_{6}$TC(TTCTC)$_{32}$C	315	
7	Parent	C(TTCTC)TT(TTCTC)$_{2}$TT(TTCTC)$_{17}$(TCTTCTC)$_{6}$TC(TTCTC)$_{25}$C	275	-1
	Offspring	C(TTCTC)TT(TTCTC)$_{2}$TT(TTCTC)$_{17}$(TCTTCTC)$_{6}$TC(TTCTC)$_{24}$C	270	

[m] Allele from same parental individual that mutated.
[n] Same mutation in Mut3 and Mut4, but in two different individuals.
